# Equivalent Gene Expression Profiles between Glatopa^™^ and Copaxone^®^

**DOI:** 10.1371/journal.pone.0140299

**Published:** 2015-10-16

**Authors:** Josephine S. D’Alessandro, Jay Duffner, Joel Pradines, Ishan Capila, Kevin Garofalo, Ganesh Kaundinya, Benjamin M. Greenberg, Daniel Kantor, Tanmoy C. Ganguly

**Affiliations:** 1 Momenta Pharmaceuticals, Inc., Cambridge, MA, United States of America; 2 The University of Texas Southwestern Medical Center, Dallas, TX, United States of America; 3 Kantor Neurology, Coconut Creek, FL, United States of America; University of Texas at San Antonio, UNITED STATES

## Abstract

Glatopa^™^ is a generic glatiramer acetate recently approved for the treatment of patients with relapsing forms of multiple sclerosis. Gene expression profiling was performed as a means to evaluate equivalence of Glatopa and Copaxone^®^. Microarray analysis containing 39,429 unique probes across the entire genome was performed in murine glatiramer acetate—responsive Th2-polarized T cells, a test system highly relevant to the biology of glatiramer acetate. A closely related but nonequivalent glatiramoid molecule was used as a control to establish assay sensitivity. Multiple probe-level (Student’s *t*-test) and sample-level (principal component analysis, multidimensional scaling, and hierarchical clustering) statistical analyses were utilized to look for differences in gene expression induced by the test articles. The analyses were conducted across all genes measured, as well as across a subset of genes that were shown to be modulated by Copaxone. The following observations were made across multiple statistical analyses: the expression of numerous genes was significantly changed by treatment with Copaxone when compared against media-only control; gene expression profiles induced by Copaxone and Glatopa were not significantly different; and gene expression profiles induced by Copaxone and the nonequivalent glatiramoid were significantly different, underscoring the sensitivity of the test system and the multiple analysis methods. Comparative analysis was also performed on sets of transcripts relevant to T-cell biology and antigen presentation, among others that are known to be modulated by glatiramer acetate. No statistically significant differences were observed between Copaxone and Glatopa in the expression levels (magnitude and direction) of these glatiramer acetate-regulated genes. In conclusion, multiple methods consistently supported equivalent gene expression profiles between Copaxone and Glatopa.

## Introduction

Copaxone^®^ (glatiramer acetate injection; Teva Pharmaceutical Industries Ltd, North Wales, PA, USA) has been approved in the United States for the treatment of relapsing forms of multiple sclerosis (MS) for nearly two decades [1,2]. Glatiramer acetate (GA) is a mixture of synthetic polypeptides of variable molecular weights and sequences and is manufactured entirely through a chemical synthesis from the amino acids L-alanine, L-glutamic acid, L-lysine, and L-tyrosine in a specific well-described molar ratio [3,4]; it is not a biologic product. Although the precise mechanism of action of GA has not been elucidated, its therapeutic actions in MS are thought to be primarily immunomodulatory [5]. These immunomodulatory effects of GA are complex and have been hypothesized to involve both the innate and adaptive immune systems [6] through various mechanisms, including alteration of regulatory T-cell function [7], induction of a T-helper 1 (Th1) to a T-helper 2 (Th2) cell shift that results in a more anti-inflammatory cytokine profile [5,8–10], alteration of antigen-presenting cell (APC) function [7], and modulation of B-cell function [6]. Additional activity of GA in MS may include neuroprotective effects mediated by neurotrophic factors and the ability to reduce demyelination and promote remyelination [6,11,12].

Increasing patient access to affordable MS medications is one of the drivers for the development of generic medicines. The first generic GA approved by the US Food and Drug Administration (FDA) is Glatopa^™^ (glatiramer acetate injection; Sandoz Inc., Princeton, NJ, USA) [13], which is indicated for the treatment of relapsing forms of multiple sclerosis. Glatopa and Copaxone equivalence was established using a comprehensive set of physicochemical (structural) and biological (functional) assays. To this end, we have developed and tested Glatopa in multiple biological assays, including APC-based, T cell-based, and B cell-based assays, and in multiple animal models of experimental autoimmune encephalitis [14,15].

One of the many methods utilized to evaluate equivalence of biological response was gene expression profiling using microarray technology. This method allows for the detection of genome-wide perturbations in a biological test system and is therefore well suited to compare drug responses [16]. We present a study of the effect of different test samples on in vitro gene expression in murine GA-responsive Th2-polarized T cells. Selection of the in vitro test system of an enriched nonclonal population of GA-responsive T cells was largely driven by two factors: Th2-polarized GA-responsive T cells are appropriate for comparative gene expression studies because they produce a robust (high signal-to-noise ratio) and reproducible (less variation) response to GA, and Th2-polarized T cells are relevant to the biology of GA given that one purported mechanism is a shift from a pro-inflammatory Th1 to an anti-inflammatory Th2 phenotype reported during prolonged exposure to GA [3,8,17]. The samples tested included a media-only control, Copaxone, and Glatopa. In addition, to establish method sensitivity, a nonequivalent glatiramoid molecule—acetonitrile nonconforming copolymer (ACN)–was also utilized. ACN was manufactured to be compositionally similar to GA but is structurally distinct. Specifically, ACN has the same general makeup as GA (polymer chains with the same molecular weight distribution and amino acid composition), but it was made by process conditions that generated a structurally nonequivalent mixture. ACN can be readily distinguished from Copaxone in some but not all physiochemical and biological assays used for establishing equivalency (data not shown).

## Materials and Methods

### Animal ethics

All animal experiments were approved and performed under the guidelines of the Institutional Animal Care and Use Committee of Momenta Pharmaceuticals Inc. (IACUC approval number 05–2011). Female Balb/c mice (8–12 weeks old) were obtained from Jackson Laboratories and used as a source for APCs or CD4^+^ T cells. The mice were allowed to eat and drink ad libitum and were fed standard mouse chow.

### Generation of Copaxone-specific Th2-polarized T cells

**Fig 1:**
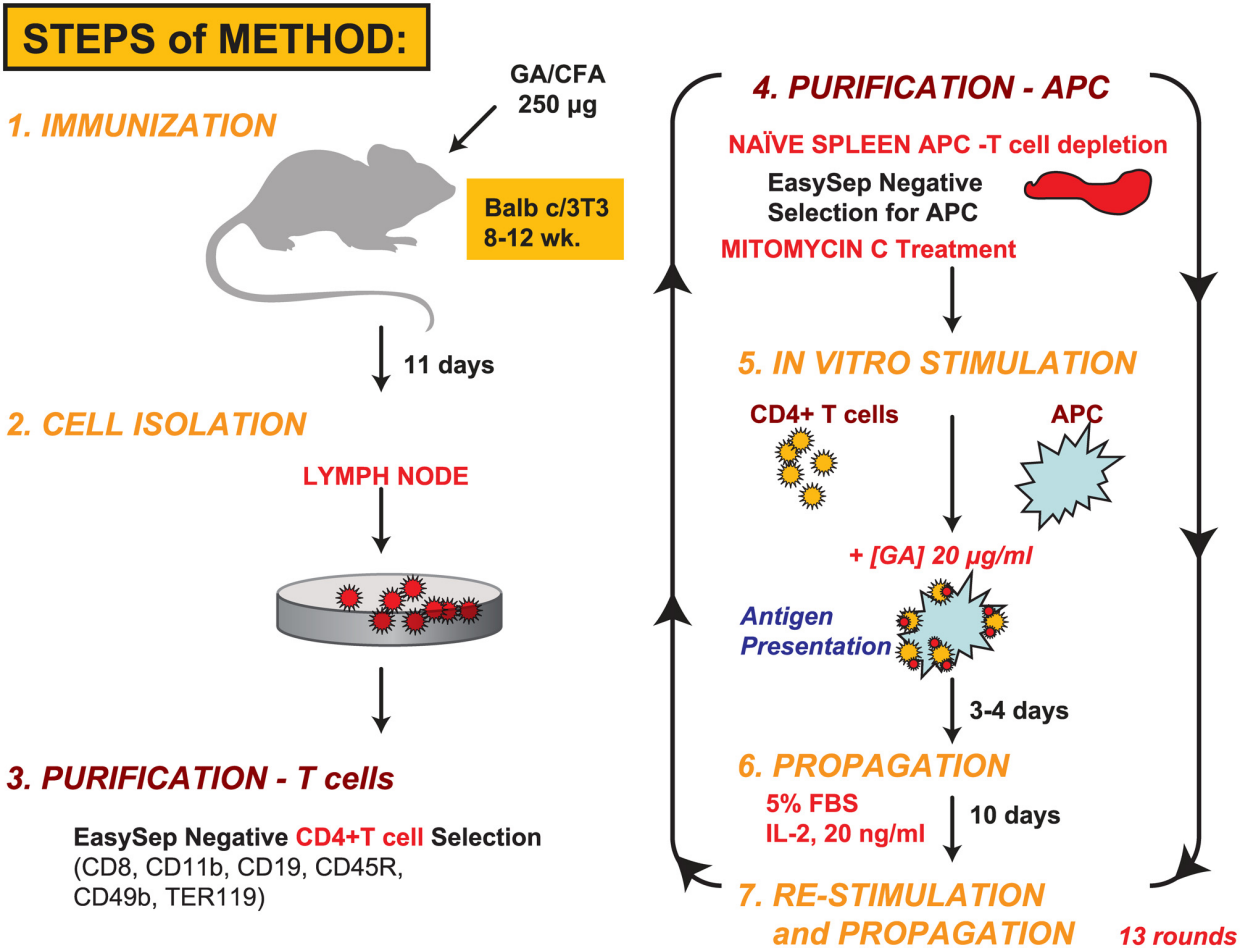
Methodology for the generation of murine GA-responsive Th2-polarized T cells. In vivo immunization of naive mice with Copaxone was followed by 13 rounds of ex vivo restimulation of the CD4^+^ T-cell population over 6 months for development of the Th2-455 line. APCs, antigen-presenting cells; GA, glatiramer acetate; IL-2, interleukin 2; Th, T-helper.

Fig 1 summarizes the steps in the generation of Copaxone-specific Th2-polarized T cells using a modification of the methods detailed in Aharoni et al [18]. Briefly, Balb/c mice were immunized with Copaxone 250 μg (lot 538455), and lymph nodes were harvested. CD4^+^ T cells were isolated from the lymph nodes using negative immunomagnetic isolation (EasySep Mouse CD4+ T Cell Isolation Kit, Stem Cell Technologies, Vancouver, BC, Canada). Splenocytes were isolated from naive (non-immunized) mouse spleens depleted of T cells using positive immunomagnetic isolation. APCs were treated with mitomycin C 50 μg/mL (Calbiochem, EMD Millipore, Billerica, MA, USA) for 25 minutes at 37°C to prevent a proliferative response from residual T cells. CD4^+^ T cells were rechallenged ex vivo for 3 to 4 days with Copaxone 20 μg/mL presented using the mitomycin C-treated splenocytes as APCs followed by a period of maintenance for 10 days in the presence of 20 ng/mL murine interleukin-2 (mIL-2; PeproTech, Rocky Hill, NJ, USA). The process of Copaxone restimulation and cell expansion followed by maintenance was repeated for 13 rounds using 20% conditioned media from the cells and mIL-2 as just described; this led to production of a Th2-polarized nonclonal T-cell population. The Th2 phenotype of these cells, which were exclusively responsive to Copaxone, was further verified by enzyme-linked immunosorbent assay (ELISA; Meso Scale Discovery, Rockville, MD, USA); mouse Th1/Th2 (9-plex); custom IL-6, IL-13, IL-17 (3-plex), and mouse TGF-β1Quantikine (R&D Systems, Minneapolis, MN, USA) ELISA kits for Th1, Th2, Th17; and other cytokines (IL-1β, IL-2, IL-4, IL-5, IL-6, IL-10, IL-12p70, IL-13, IL-17, tumor necrosis factor-alpha [TNF-α], and transforming growth factor-beta 1 [TGF-β1]). These cells exhibited Copaxone-induced dose-dependent release of Th2-specific cytokines (IL-4, IL-5, IL-10, and IL-13), whereas levels of Th1 cytokines (TNF-α and interferon-gamma [IFN-γ]) were negligible. The cell bank of Th2-polarized T cells (designated Th2-455) was created and used for all gene expression studies.

### Test samples

Three types of test samples were examined: nine lots of Copaxone tested in duplicate (lot numbers P53856, P53835, P53853, X05691, X05781, X05831, X05751, X06841, and X06661), four lots of Glatopa tested in quadruplet (lot numbers 051M7282, 061M7276, 071M7276, and 100M7278), and one lot of the nonequivalent glatiramoid, (ACN) tested in eight replicates (lot number FA0907-051-001). The number of samples provided adequate power for statistical comparison of Copaxone (17 samples) to Glatopa (16 samples) via permutation testing, while also capturing the diversity of Copaxone and Glatopa lots.

### In vitro stimulation with test samples

The concentrations of all test articles were adjusted based on A275-nm measurements. The sample order was randomized and blinded before initiation of the experiment to minimize bias. All samples were tested on the same day to minimize the introduction of bias resulting from sample processing. First, APC 0.5 mL (naive Balb/C splenocytes, ReachBio LLC, Seattle, WA, USA) cell suspension (10 × 10^6^ cell/mL) was added to each well of a 24-well plate, followed by Th2-455 cell suspension 0.5 mL (2 × 10^6^ cell/mL). Then 10 μL media or test article was added for a final concentration of 20 μg/mL. The plates were incubated for 24 hours at 37°C in a 5% CO_2_ incubator. All cells, both in suspension and adherent, were harvested and spun at 800*g* at 4°C for 5 minutes. The conditioned media were removed, and 0.35 mL RLT lysis buffer was immediately added to the cell pellet that was then solubilized and frozen at -80°C until RNA purification.

### Cell lysis and RNA purification

RNA processing was performed by blinded operators distinct from those involved in the in vitro stimulation step described in the previous section, and was performed at the same time to minimize the introduction of bias resulting from sample processing. Lysates were thawed and passed through a QiaShredder (Qiagen Inc, Valencia, CA, USA) column. RNA was extracted using the RNEasy standard protocol on the QIACube (Qiagen Inc) instrument. RNA was eluted in 50 μL distilled water. RNA concentration was measured by ultraviolet light, and integrity was measured by Bioanalyzer Nano chip (Agilent Technologies, Wilmington, DE, USA). Only RNAs of acceptable quality (28S/18S ratio close to 2) were subsequently processed.

### Whole-genome microarray analysis

Gene expression was analyzed by oligonucleotide microarrays using the Mouse GE 4x44K v2 Microarray Kit (Agilent Technologies), containing probes specific for murine transcripts. This microarray platform has four arrays printed on each slide each of which contains 39,429 unique probes that cover the entire mouse genome. To minimize the introduction of bias, microarrays from the same lot were used, and all sample processing was performed in one batch. A total of 48 samples were used for microarray analysis: media-only control, *n* = 7; Copaxone, *n* = 17; Glatopa, *n* = 16; and ACN, *n* = 8. Data from two arrays—one replicate of Copaxone lot X06841 and one media-only sample—were excluded from analysis because of poor array quality.

Sample RNA was first normalized to obtain 99 ng per reaction and then mixed with a spike-in control (5188–5282; Agilent Technologies). Cy3-labeled cRNA was then generated by in vitro transcription (5190–2305; Agilent Technologies), and the concentration was adjusted to 1.65 μg per slide. Microarrays were placed into the Tecan HS Pro 4800 (Tecan Group Ltd, Männedorf, Switzerland) hybridization station, the hybridization program was started, and labeled RNA was introduced into the chambers. After hybridization and washing, the slides were dried and scanned using the GenePix scanner (Axon Instruments Inc, Foster City, CA, USA). The gene array list file was then fit to the slide images in the GenePix Pro software (Axon Instruments Inc). Fluorescence foreground and background were extracted for each spot.

### Raw data preparation and statistical analysis

Statistical analyses were performed using the open-source statistical environment R [19]. Median fluorescence data were loaded into R for analysis using the *limma* package [20]. The log2-transformed intensities were background-corrected using the “normexp” method [21] with an offset of 8 and were normalized using the quantile normalization method [22]. All microarray data are in accordance with the MIAME guideline and are accessible through a Gene Expression Omnibus (GEO) database (Accession number GSE73465, http://www.ncbi.nlm.nih.gov/geo/query/acc.cgi?acc=GSE73465). The quantile-normalized, normexp background—corrected, replicate probe—averaged data were analyzed at both the sample level and the probe level using multiple (multivariate and univariate) statistical analysis methods, as described in the following two sections.

### Sample-level analysis

Several multivariate statistical methods were used to analyze the dataset. Multivariate analyses were performed not only with a subset of Copaxone-responsive probes (4176 array spots, which yielded a q-value [21] <0.05 and fold change of ≥1.3 when comparing Copaxone with media-treated samples) but also with all transcripts. The assessment of significance of observed multivariate statistics was based on a null model of permutation control (i.e., observed values compared with those expected for samples randomly assigned to groups).

Principal component analysis (PCA) is a statistical technique whereby correlated dimensions within a dataset are transformed into a set of linearly uncorrelated dimensions [23]. Before PCA, data from each microarray probe were mean centered and scaled by dividing by the standard deviation among all samples. PCA was performed on the entire set of data using the prcomp function of the R stats package. The first component was extracted to visualize differences among groups of samples in the data set. t-Tests with Welch correction were used to check for evidence of differences between groups.

Multidimensional scaling (MDS) is a method that provides visual representation of distances between high-dimensional objects, such as individual samples (vectors of array spot intensities). MDS represents samples as points in a plane; point positions are optimized so as to reflect distances between samples [24], and distance is defined here with Pearson dissimilarity.

Unsupervised hierarchical clustering is a commonly used data technique in bioinformatics wherein objects (samples) are grouped together based on a measure of distance or dissimilarity between objects. The “hclust” function of R, based on Ward’s measure, was used [19]. This greedy agglomerative clustering method creates a binary tree by using Pearson dissimilarity matrix as an input.

In addition, a statistic based on sample Pearson dissimilarities (*d*_*ij*_) was defined as follows to compare any two groups G and H of samples:

$$\;mvt=\;\frac{d\left(G,H\right)}{s\left(G\right)+s\left(H\right)}\text{ with }d\left(G,H\right)=\frac{1}{\left|G\right|\left|H\right|}\sum_{i\in G,j\in H}d_{ij}\text{ and }s\left(G\right)=\frac{2}{\left|G\right|\left(\left|G\right|-1\right)}\sum_{i<j\in G}d_{ij}.$$

This statistic is referred to as multivariate statistic t (mvt) due to its similarity to the univariate t statistic: ratio of difference between two groups to spread within individual groups. Observed values of mvt were compared with values MVT expected when randomly reassigning samples between the two groups; that is, Pr(MVT >mvt) was estimated with 10^5^ permutations.

### Transcript- and probe-level analysis

Differential expression between two test materials in the Th2-polarized T-cell test system was examined by pairwise t-test at the level of gene expression microarray probes. Three different methods were used to identify significant changes. First, the Bonferroni correction method was used to set a *P*-value threshold for significance. The Bonferroni correction is a familywise error rate (FWER) method that controls for the number of false positives and assumes that gene transcript expression for individual transcripts is independent [25]. It sets a conservative threshold for ensuring that probes that pass this threshold have an extremely small chance of being the result of random variation. The *P*-value threshold resulting from the Bonferroni correction is *P*_i_ < α/*n*, where α = 0.05 and *n* is the number of probes (39,429) on the microarray.

Second, the false discovery rate (FDR) correction [26] was implemented through the q-value package in R [19]. The FDR was set to *q* = 0.05, and the smoother method was used for estimating the number of true null hypotheses. Unlike the Bonferroni correction, which controls for the number of allowed false positives, the FDR controls for the proportion of false positives in a given set of data [27]. As with the Bonferroni method, this FDR method operates under the assumption of independence of expression of different transcripts. However, because expression of individual gene transcripts is not always independent, observed numbers of genes with small corrected *P*-values were also compared with those expected under a null model of permutation control, which preserves gene coexpression.

Third, the permutation control method gauges whether the number of probes less than a given *P*-value threshold is more than expected by chance [28]. Samples from two groups to be compared were randomly reassigned to two groups with proportions reflecting the original groups. Student’s t-tests were performed, and the number of probes with *P*-values less than a given threshold was computed; 2000 permutations were performed. The sum of the permutations for which the number of significantly different probes was less than that found in the original data was computed. A permutation *P*-value was calculated where this sum was divided by the total number of permutations. A permutation *P* < 0.05 indicated that there were more significantly different probes in the original data than would be expected by chance. Together, these methods probed for any statistically significant differences in gene expression between test materials.

### Ingenuity pathway analysis

Molecular pathways involved in collections of microarray probes were examined with the Ingenuity Pathway Analysis program (Qiagen Inc). This program integrates relevant information from imported genes, with built-in pathways for T cell and APC biology, consequently allowing for identification of biological pathways, gene regulation networks, and interaction maps.

## Results

### Copaxone-induced gene expression changes in murine GA-responsive, Th2-polarized T cells

The methodology used to create the GA-responsive, Th2-polarized T cell line (Th2-455) in Balb/c mice used in these studies is illustrated in Fig 1. Briefly, after in vivo immunization with Copaxone, CD4-purified T cells were expanded by ex vivo GA restimulation for 13 rounds and then frozen to create a cell bank used for the gene expression studies.

**Fig 2:**
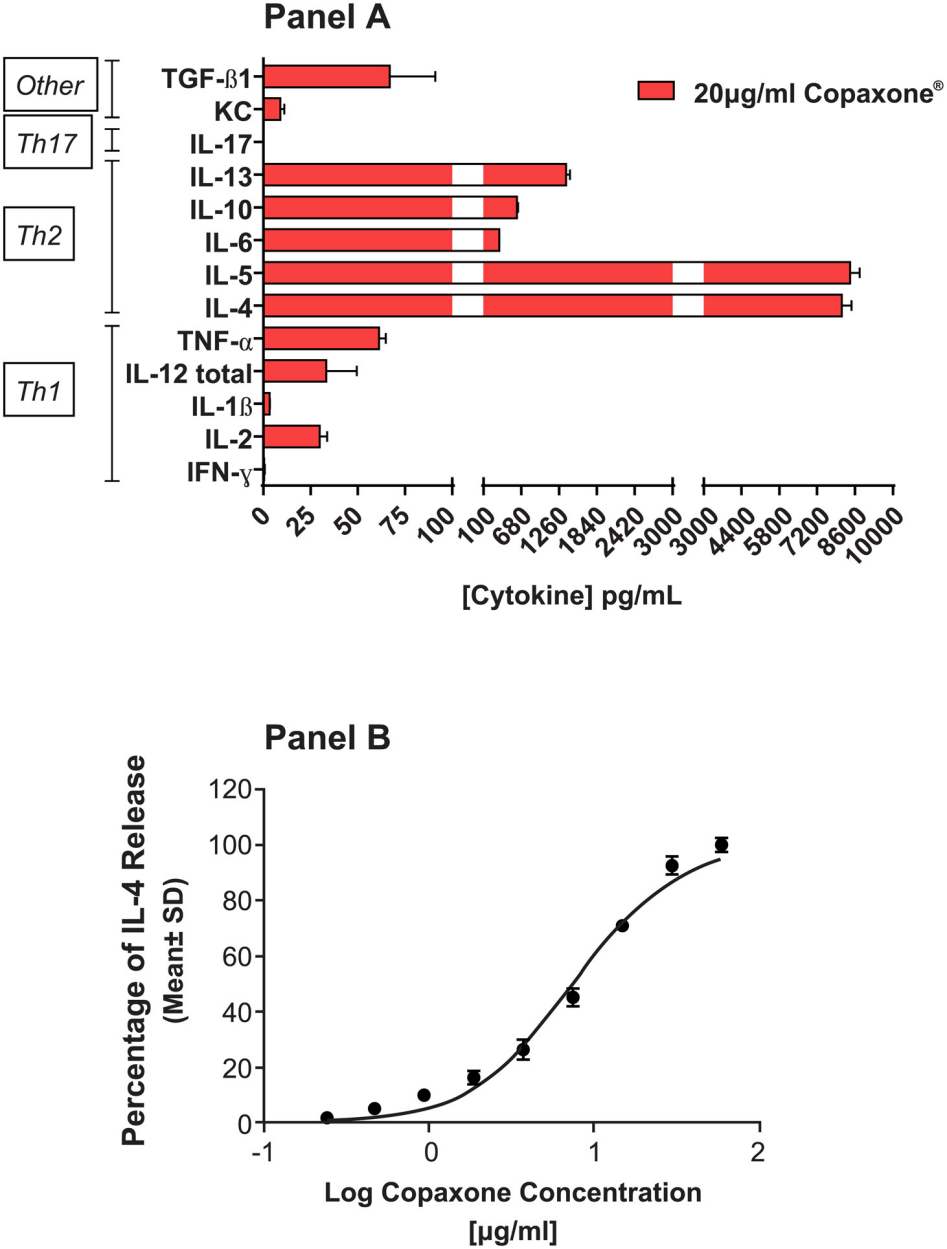
Characterization of the murine Th2-polarized T cells. Cytokine profile and dose response. **A:** Cytokine profile of the conditioned medium from the Th2-455 cell line after 24 hours of treatment with Copaxone at a single concentration of 20 μg/mL, demonstrating Th2 polarization. **B:** Copaxone dose response at 24 hours of a single cytokine, IL-4. IFN-γ; interferon gamma; KC, keratinocyte chemoattractant; IL, interleukin; TGF-β1, transforming growth factor-beta 1; Th, T-helper.

The phenotype of the GA-responsive murine Th2 T cells was confirmed by single and multiplexed ELISAs for Th1 and Th2 cytokines. These cells secreted low levels of the Th1 cytokines (IL-1β, TNF-α, and IFN-γ), whereas the Th2 cytokines (IL-4, IL-5, IL-10, and IL-13) were released at high concentrations (Fig 2A). Copaxone produced a dose-dependent increase in IL-4 release from these Th2-polarized T cells (Fig 2B); a similar dose effect was seen with the other cytokines (data not shown). Whole-genome gene expression of multiple samples of Copaxone, in 17 samples from 9 different lots, were compared with those of 7 samples of media-only controls. The number of probes that were significantly different was analyzed using univariate analysis (Student’s t-test). The results of univariate analysis (Table 1) indicated that of the 39,429 probes evaluated at the probe level by pairwise comparison, 9815 probes were significantly different at a *P*-value threshold of 0.05 (before controlling for false positives) when comparing Copaxone with media-only control. Evaluation of the data using FDR (threshold q = 0.05) or the more conservative FWER with Bonferroni correction test indicated that 6869 and 1080 probes, respectively, were significantly different in Copaxone than in the media-only control. Permutation control analysis on this data set indicated that these differences were not attributed to random sampling of the data, as indicated by the significance (*P* < 0.0005) of observing the given number of probes compared with the null hypothesis of no difference between groups.

**Table 1:** Number of different probes between Copaxone, Glatopa, nonequivalent glatiramoid molecule ACN, and media (univariate analysis, *P* < 0.05*).

Sample	Significantly different probes, before controlling for false positives, n	Significantly different probes (using FDR at q = 0.05), n	Significantly different probes (using FWER with Bonferroni correction), n	Significance by permutation control (*P*-value)
Copaxone vs media-only control	9815	6869	1080	0.0005
Copaxone vs Glatopa	1626	0	0	0.753
Copaxone vs ACN	3100	11	3	0.0240

FDR, false discovery rate; FWER, familywise error rate.

*Student’s t-test.

These observations provide evidence for gene expression alterations induced by Copaxone relative to the media-only control, thus providing the foundation and scientific rationale for the use of this biological test system to compare the effects of Glatopa on gene expression.

### Comparison of differences of induced gene expression between Copaxone and Glatopa or ACN

The same three univariate statistical methods used to compare Copaxone with media-only control were used to examine differences between Copaxone and multiple Glatopa samples (16 samples from four lots) and between Copaxone and ACN (eight samples from one lot). Results of this analysis are also summarized in Table 1. Comparison of Glatopa samples and Copaxone showed 1626 probes with *P* < 0.05, before controlling for false positives. Permutation testing indicated that the number of differences observed in the original data was not significantly higher than those observed in randomly scrambled data (P = 0.753). After controlling for false positives, both the FDR at q = 0.05 and FWER using Bonferroni correction at *P* = 0.05 showed that there were no differentially expressed genes between these two groups; thus, Glatopa and Copaxone were equivalent for gene expression changes.

Comparison of gene expression profiles of Copaxone and ACN yielded 3100 probes that were significantly different (P<0.05). Control of false positives using FDR resulted in 11 probes with a significant difference between Copaxone and ACN (the corresponding *P* value to q = 0.05 was *P* = 0.0000139), and three probes exhibited a significant difference between Copaxone and ACN using a Bonferroni-corrected *P*-value threshold for control by FWER. Furthermore, the less conservative approach based on permutation testing also indicated significance (*P* = 0.024) when comparing Copaxone and ACN, thereby suggesting that these differences were not caused by random chance.

In summary, these univariate analyses of the probe-level data did not reveal any significant differences in the gene expression between Copaxone and Glatopa; however, a subset of genes was significantly different between Copaxone and ACN, underscoring the sensitivity of the test system and the method of analysis.

### Multivariate statistical analysis

Multivariate analyses were first performed based on a subset of 4176 probes that were found to be Copaxone responsive (q-value less than 0.05 and fold change of at least 1.3 when comparing Copaxone and media groups). Other treatment groups were then compared with these 4176 Copaxone-responsive probes for their ability to yield probes with q-values less than 0.05 and fold change greater than 1.3; the results are presented in Table 2. Glatopa yielded a very similar comparison to media to that obtained with Copaxone: 3753 probes led to q-values less than 0.05 and fold change over 1.3. The nonequivalent glatiramoid control ACN also gave a significant difference to media with 2608 probes. The response to ACN was also found to be significantly different than the responses to Copaxone (66 probes) and Glatopa (153 probes), yet none of the Copaxone-responsive probes were found to be significantly different when comparing Glatopa with Copaxone. These results show that the Copaxone-response signature of 4176 probes can significantly distinguish between GA (Copaxone or Glatopa) and a nonequivalent glatiramoid control (ACN), yet does not yield any significant difference between Copaxone and Glatopa.

**Table 2:** Number of significantly perturbed probes (q-value < 0.05 and fold change > 1.3) when comparing sample groups. An initial set of 4176 probes was detected by comparing Copaxone and media groups. This set yielded significant differences between Glatopa and media and between nonequivalent glatiramoid ACN and glatiramer acetate (Copaxone or Glatopa), but no significant difference between Copaxone and Glatopa.

	Copaxone	Glatopa	ACN	Media
Copaxone	—	0	66	4176
Glatopa	0	—	153	3753
ACN	66	153	—	2608
Media	4176	3753	2608	—

**Fig 3:**
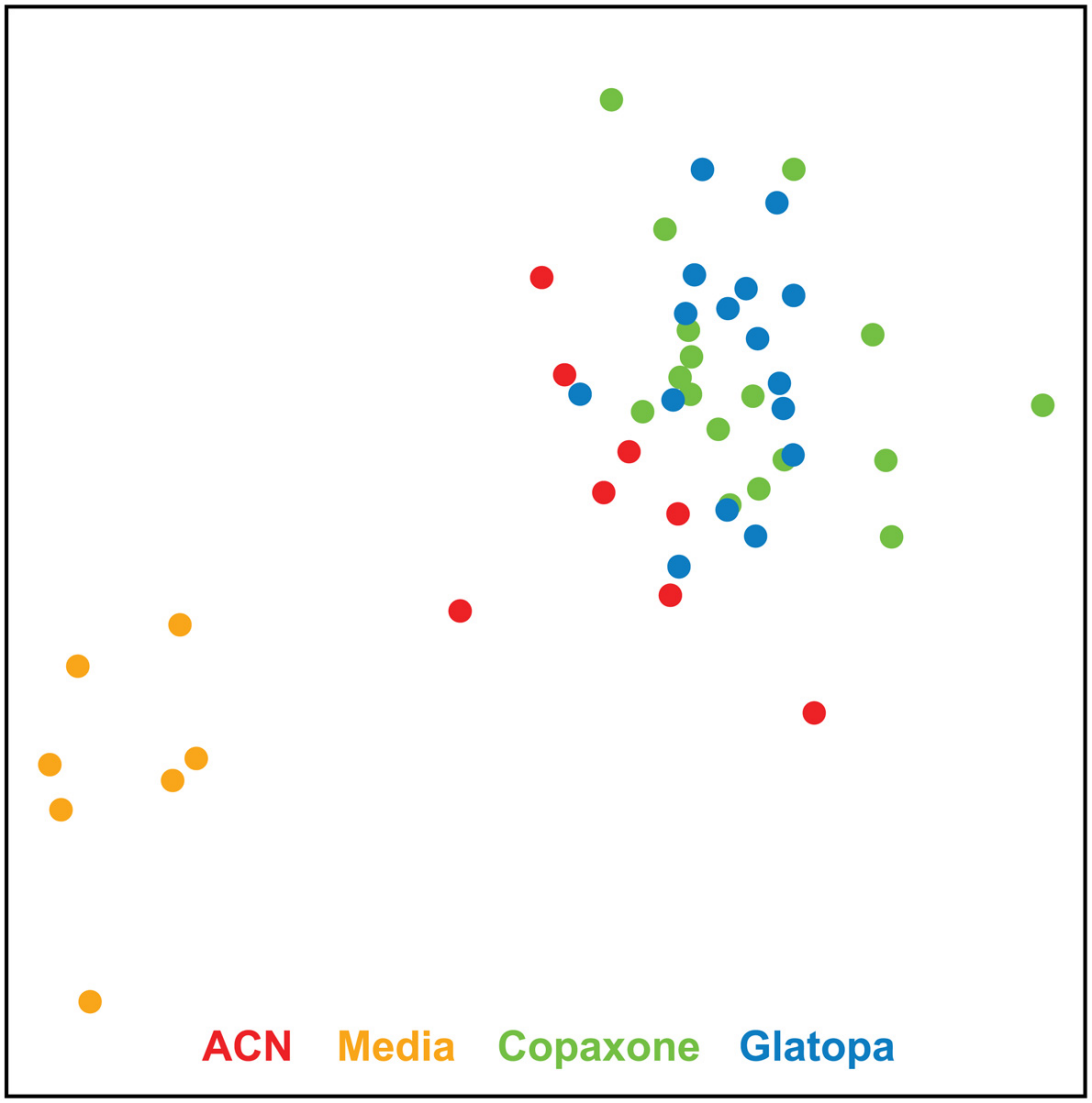
MDS plots based on the 4176 Copaxone-responsive probes. As expected, clear separation is obtained between Copaxone and media groups. There is also clear separation between media and copolymer groups (ACN, Copaxone, or Glatopa), some separation between GA (Copaxone or Glatopa) and ACN and no visible separation between Copaxone and Glatopa. ACN, acetonitrile nonconforming copolymer; GA, glatiramer acetate; MDS, multidimensional scaling.

The results presented in Table 3, which are based on multivariate statistic t, corroborated results of Table 2. Based on permutation testing, which preserves gene coexpression, Table 3 showed a significant difference between Glatopa and media, a significant difference between ACN and media, a significant difference between GA (Copaxone or Glatopa) and ACN, and no significant difference between Copaxone and Glatopa (*P* = 0.5). The MDS plot (Fig 3) provides a visual illustration of these conclusions based on the 4176 Copaxone-responsive probes. There is clear separation between media and the three other groups (ACN, Glatopa, and Copaxone) and separation between ACN and GA (Copaxone or Glatopa), but there is no meaningful separation between Copaxone and Glatopa.

**Table 3:** *P*-values for multivariate statistic t inferred by permutation control (10^5^ random assignments of samples between compared groups) based on the 4176 Copaxone-responsive probes. In agreement with results presented in Table 2, there were significant differences when comparing Copaxone with media, Glatopa with media, nonequivalent glatiramoid ACN with media, and ACN with glatiramer acetate (Copaxone or Glatopa), but no significant difference for Copaxone vs Glatopa.

	Copaxone	Glatopa	ACN	Media
Copaxone	—	0.5	0.0009	0.0001
Glatopa	0.5	—	0.0001	0.0001
ACN	0.0009	0.0001	—	0.001
Media	0.0001	0.0001	0.001	—

**Fig 4:**
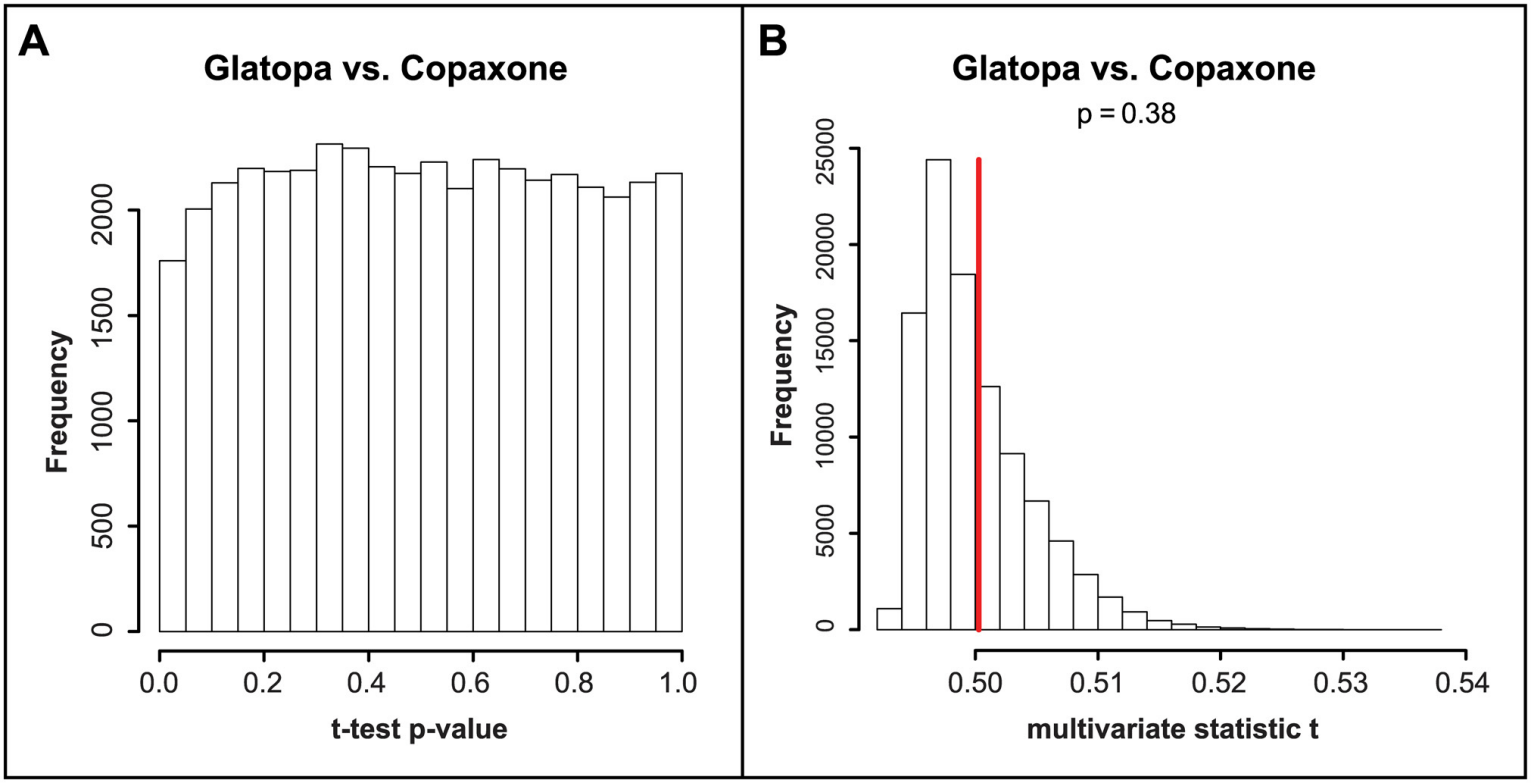
Statistical comparisons between Copaxone and Glatopa based on all probes. **A:** distribution of t-test *P*-values across all array spots. The proportion of small *P*-values (*P* < 0.05) is small and the largest obtained q-value is greater than 0.99. **B:** multivariate statistic t (MVT) results. The observed value of MVT for Copaxone vs Glatopa is not significantly large as compared to values expected when randomly mixing samples between these two groups (*P* = 0.38).

**Fig 5:**
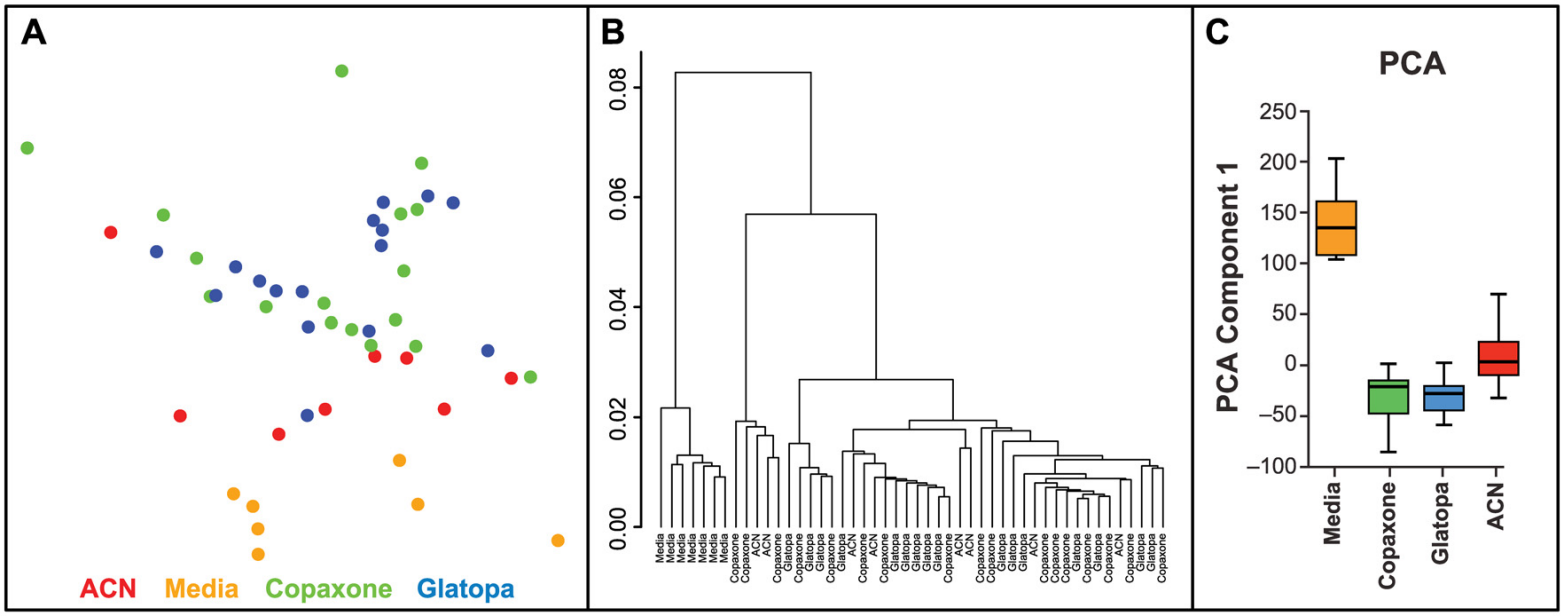
Visual comparisons between Copaxone, Glatopa and ACN treatment groups based on all probes. **A:** The MDS plot shows separation between glatiramer acetate (GA; Copaxone or Glatopa) and media, separation between ACN and media, and no separation between Copaxone and Glatopa. **B:** Hierarchical clustering yields separation between GA (Copaxone or Glatopa) or ACN and media and no separation between Copaxone and Glatopa. **C:** Box plot of principal component analysis (PCA) component 1. Includes samples from media control, Copaxone, Glatopa, and ACN.

Results presented in Tables 2 and 3 demonstrate that gene expression responses to Copaxone and Glatopa were not significantly different even though probes utilized to test for such response are sensitive enough to distinguish between GA (Copaxone or Glatopa) and a nonequivalent glatiramoid (ACN). To investigate whether any gene expression differences outside of Copaxone response might exist between Copaxone and Glatopa, all transcripts were considered rather than only those responsive to Copaxone. Statistical results are presented in Fig 4. Fig 4A shows that there is no overrepresentation of small t-test *P*-values when comparing Copaxone and Glatopa, and this was confirmed by no q-value less than 0.99 for this group comparison. A more stringent statistical evaluation, one which takes into account coexpression between genes, was obtained by using multivariate statistic t; Fig 4B shows no statistically significant difference between Copaxone and Glatopa based on all probes (*P* = 0.38). Fig 5 provides visual illustrations for the lack of significant difference between Copaxone and Glatopa based on all probes. The MDS plot (Fig 5A) shows clear separation between media and GA (Copaxone or Glatopa) but no separation between Copaxone and Glatopa, while samples corresponding to ACN treatment tend to fall between media and GA samples. Likewise, hierarchical clustering (Fig 5B) yields clear separation between media and GA but no obvious separation between Copaxone and Glatopa. Unlike MDS, hierarchical clustering does not suggest separation of ACN samples from GA-treated samples. Fig 5C shows the results of PCA. The media-only control groups were well separated from the GA groups. Comparison of the mean level of principal component 1 between the media-only control group and each treatment group indicated *P* < 0.001 (t-test). Copaxone and Glatopa could not be distinguished from each other (*P* = 0.911). To summarize the findings of multiple multivariate statistical analysis methods, using appropriate permutation control, there were no significant difference between Copaxone and Glatopa; however, these same analysis methods indicated differences between Copaxone and ACN.

### Comparative analysis across transcripts relevant to known biological action of GA

Multiple types of statistical analyses showed that Copaxone and Glatopa were indistinguishable in the test system using Th2-polarized T cells. Therefore, for additional analysis, we focused on sets of transcripts relevant to antigen presentation and T-cell biology and relevant to the current consensus on the mechanisms of action of GA in the treatment of MS related to these two immune system cell types.

**Fig 6:**
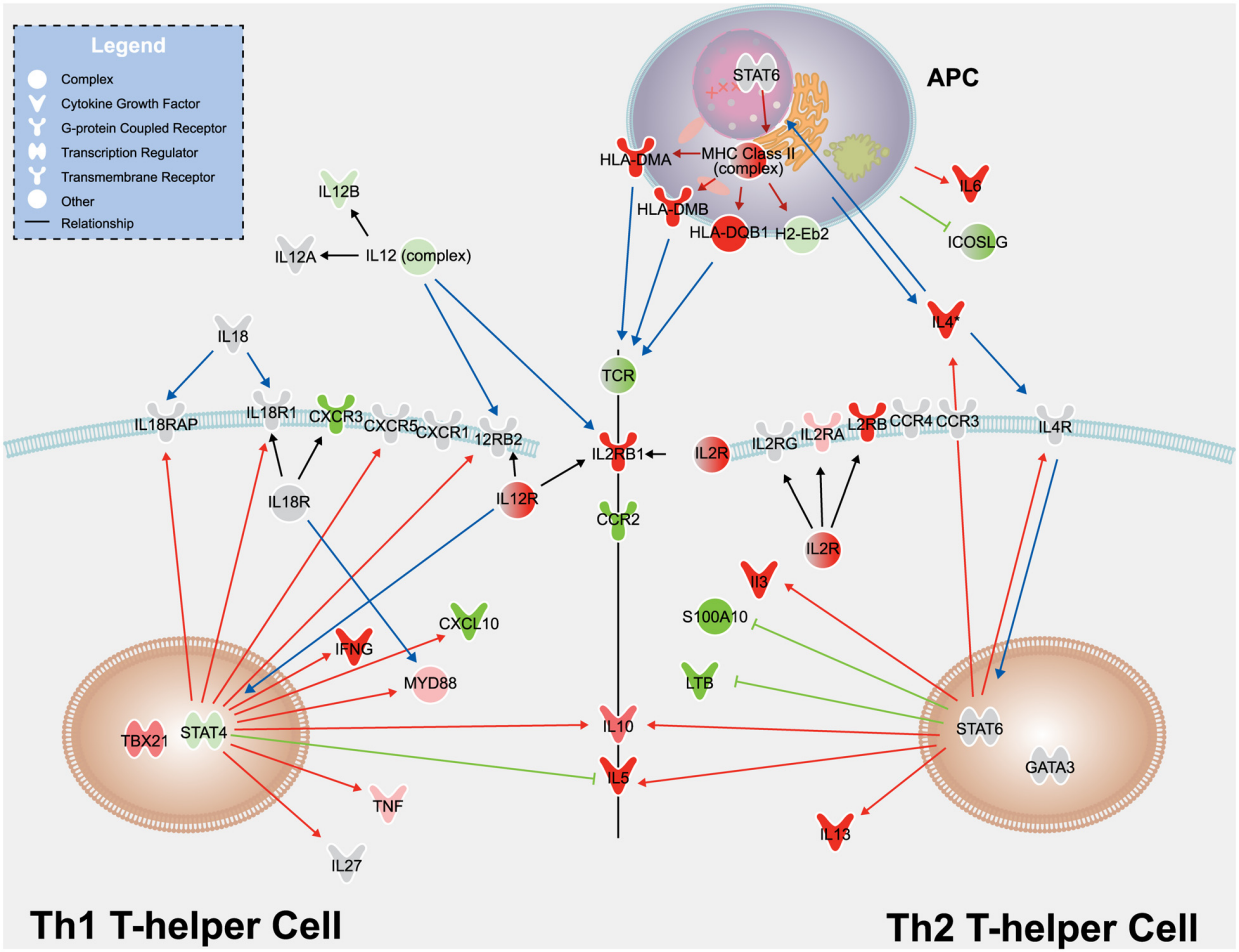
T-helper cell pathway diagram. Transcripts measured in the current study are shown in the diagram as nodes and are colored based on the differences observed when cells are stimulated with Copaxone in comparison with cell culture media alone (*P* < 1e-3 [Student’s t-test]; red for increase, green for decrease). Molecules with *P* > 1e-3 are shown in gray. Genes refer to the human ortholog. Human HLA-DMA, HLA-DMB, and HLA-DQB1 represent murine H2-Dma, H2-Dmb2, and H2-Ab1, respectively. Blue arrows show the flow of activation by major Th1- and Th2-influencing molecules in the pathway. For example, APCs produce IL-4, which binds to and activates the IL-4 receptor, leading to the phosphorylation and activation of the transcription factor STAT6. Red and green arrows show the expected transcriptional outcomes of Th1 and Th2 polarization; red and green arrows indicate that activation will cause the transcript to increase and decrease, respectively. Expected transcriptional outcomes are based on reports in the literature on Th1/Th2 T-cell polarization and on studies conducted with GA [3,4,8,10,17,29–34]. Gray lines indicate members of a group. APCs, antigen-presenting cells; CXCR1, chemokine (C-X-C motif) receptor 1; CXCR3, chemokine (C-X-C motif) receptor 3; CXCR5, chemokine (C-X-C motif) receptor 5; CXCL10, chemokine (C-X-C motif) ligand 10; GA, glatiramer acetate; HLADMA, major histocompatibility complex class II, DM alpha; HLADMB, major histocompatibility complex class II, DM beta; HLADQB1, major histocompatibility complex class II, DQ beta 1; ICOSLG, inducible T-cell costimulatory ligand; IL-4, interleukin-4; MHC class II, major histocompatibility complex class II; LTB, lymphotoxin beta (tumor necrosis factor superfamily, member 3); MYD, myeloid differentiation primary response protein; S100A10, S100 calcium-binding protein A10; STAT, signal transducer and activator of transcription; TCR, T-cell receptor; Th, T-helper.

T-helper cell differentiation can be induced by T-cell receptor stimulation by APCs. In our study, APCs (naive splenocytes) were mixed with T cells derived from GA-immunized mouse lymph nodes to provide T-cell receptor stimulation. APCs process the GA copolymer, load major histocompatibility complex II (MHC II) receptors with peptide fragments, and present these peptides to T cells. In the context of T-cell stimulation and differentiation, several cytokines can induce T-cell polarization toward a Th2 or a Th1 phenotype. Fig 6 shows a pathway diagram of Th1 and Th2 T-cell polarization, created using literature surrounding T-helper cell differentiation and studies with GA stimulation of T-cell populations.[3,4,8,10,17,29–34]. T-helper cells differentiate toward a Th2 phenotype and produce a Th2 response in the presence of the cytokine IL-4 and T-cell receptor stimulation by MHC II molecules. Th2 cells themselves also produce IL-4. In our study, we observed increased IL-4 expression when cells were stimulated with GA. IL-4 signals through its cognate IL-4 receptor, activating the JAK/STAT pathway, specifically STAT6. STAT6 is regulated by phosphorylation in the cytoplasm, then translocates to the nucleus to induce activation of another transcription factor, GATA3. Consistent with posttranslational regulation by the phosphorylation of STAT6 and GATA3, no changes were observed in gene expression of these signaling molecules. These transcription factors, however, can induce and inhibit an array of genes further downstream of the signaling cascade. As expected, STAT6/GATA3 induced increases in expression of the Th2 cytokines IL-3, IL-4, IL-5, and IL-13; the MHC II family genes; and IL-4R. Similarly, decreases were observed in the expression of other STAT6/GATA3-regulated genes, such as S100A10, ICOSLG, and LTB. This subset of genes can be considered GA treatment-specific markers for the Th2-polarized T-cell test system, and our observation of changes in the gene expression of these markers is consistent with the literature regarding Th2 cells and the purported mechanisms of GA on immune cells [3,4,7,8,10,17,29–34].

To complement the earlier analysis (Table 1) on changes to global gene expression profiling, the expression levels for these specific marker genes were compared for Copaxone, Glatopa, and the media-only control. Table 4 depicts the log2-fold change for Copaxone and Glatopa compared with media for a subset of microarray probes corresponding to the marker genes highlighted as nodes in the pathway diagram. Copaxone and Glatopa showed similar directional fold changes in gene expression compared with the media-only control and were not distinguished from each other when a subset of genes was evaluated. Table 5 lists *P*-values and log2-fold differences for Glatopa compared with Copaxone.

**Table 4:** Gene expression levels in Th2-polarized cells exposed to Copaxone and Glatopa vs media alone.

Symbol	Gene Name	Copaxone log_2_ fold difference*	Glatopa log_2_ fold difference*
**CCR2**	Chemokine (C-C motif) receptor 2	–0.7	–0.7
**CXCL10**	Chemokine (C-X-C motif) ligand 10	–0.8	–0.8
**CXCR3**	Chemokine (C-X-C motif) receptor 3	–0.6	–0.6
**H2-Eb2**	Histocompatibility complex class II antigen E beta 2	–0.3	–0.3
**HLADMA**	Major histocompatibility complex class II, DM alpha	0.8	0.8
**HLADMB**	Major histocompatibility complex class II, DM beta	1.0	0.9
**HLADQB1**	Major histocompatibility complex class II, DQ beta 1	0.6	0.7
**ICOSLG**	Inducible T-cell costimulatory ligand	–1.9	–1.9
**IL-10**	Interleukin-10	0.7	0.8
**IL-12RB1**	Interleukin-12 receptor, beta 1	1.1	1.2
**IL-13**	Interleukin-13	4.1	4.0
**IL-2RA**	Interleukin-2 receptor, alpha	0.6	0.7
**IL-2RB**	Interleukin-2 receptor, beta	1.0	1.2
**IL-3**	Interleukin-3	3.7	3.9
**IL-4**	Interleukin-4	2.4	2.6
**IL-5**	Interleukin-5 (colony-stimulating factor, eosinophil)	2.4	2.5
**IL-6**	Interleukin-6 (interferon, beta 2)	1.1	1.3
**LTB**	Lymphotoxin beta (tumor necrosis factor superfamily, member 3)	–1.0	–1.1
**S100A10**	S100 calcium-binding protein A10	–1.3	–1.2

Th, T-helper.

*Log2 of the fold difference in mean expression for the indicated test material compared with media alone.

**Table 5:** Gene expression levels in Th2-polarized cells exposed to Glatopa vs Copaxone.

Symbol	Gene Name	Glatopa *P*-value*	Log_2_ Fold difference vs Copaxone†
**CCR2**	Chemokine (C-C motif) receptor 2	0.473896	0.1
**CXCL10**	Chemokine (C-X-C motif) ligand 10	0.956119	0.0
**CXCR3**	Chemokine (C-X-C motif receptor 3	0.360223	–0.1
**H2-Eb2**	Histocompatibility 2 class II antigen E beta 2	0.768097	0.0
**HLADMA**	Major histocompatibility complex class II, DM alpha	0.977577	0.0
**HLADMB**	Major histocompatibility complex class II, DM beta	0.33147	–0.1
**HLADQB1**	Major histocompatibility complex class II, DQ beta 1	0.381386	0.1
**ICOSLG**	Inducible T-cell costimulatory ligand	0.608632	0.0
**IL-10**	Interleukin-10	0.018739	0.1
**IL-12RB1**	Interleukin-12 receptor, beta 1	0.152837	0.1
**IL-13**	Interleukin-13	0.928521	0.0
**IL-2RA**	Interleukin-2 receptor, alpha	0.057472	0.2
**IL2RB**	Interleukin-2 receptor, beta	0.168497	0.2
**IL-3**	Interleukin-3	0.025309	0.2
**IL-4**	Interleukin-4	0.002263	0.2
**IL-5**	Interleukin-5 (colony-stimulating factor, eosinophil)	0.292582	0.2
**IL-6**	Interleukin-6 (interferon, beta 2)	0.067614	0.1
**LTB**	Lymphotoxin beta (tumor necrosis factor superfamily, member 3)	0.380219	–0.1
**S100A10**	S100 calcium-binding protein A10	0.158363	0.1

Th, T-helper.

*P-values are from Student’s t-tests comparing the mean expression of Glatopa with that of Copaxone.

^†^Log2 of the fold difference in mean expression for Glatopa compared with Copaxone.

**Fig 7:**
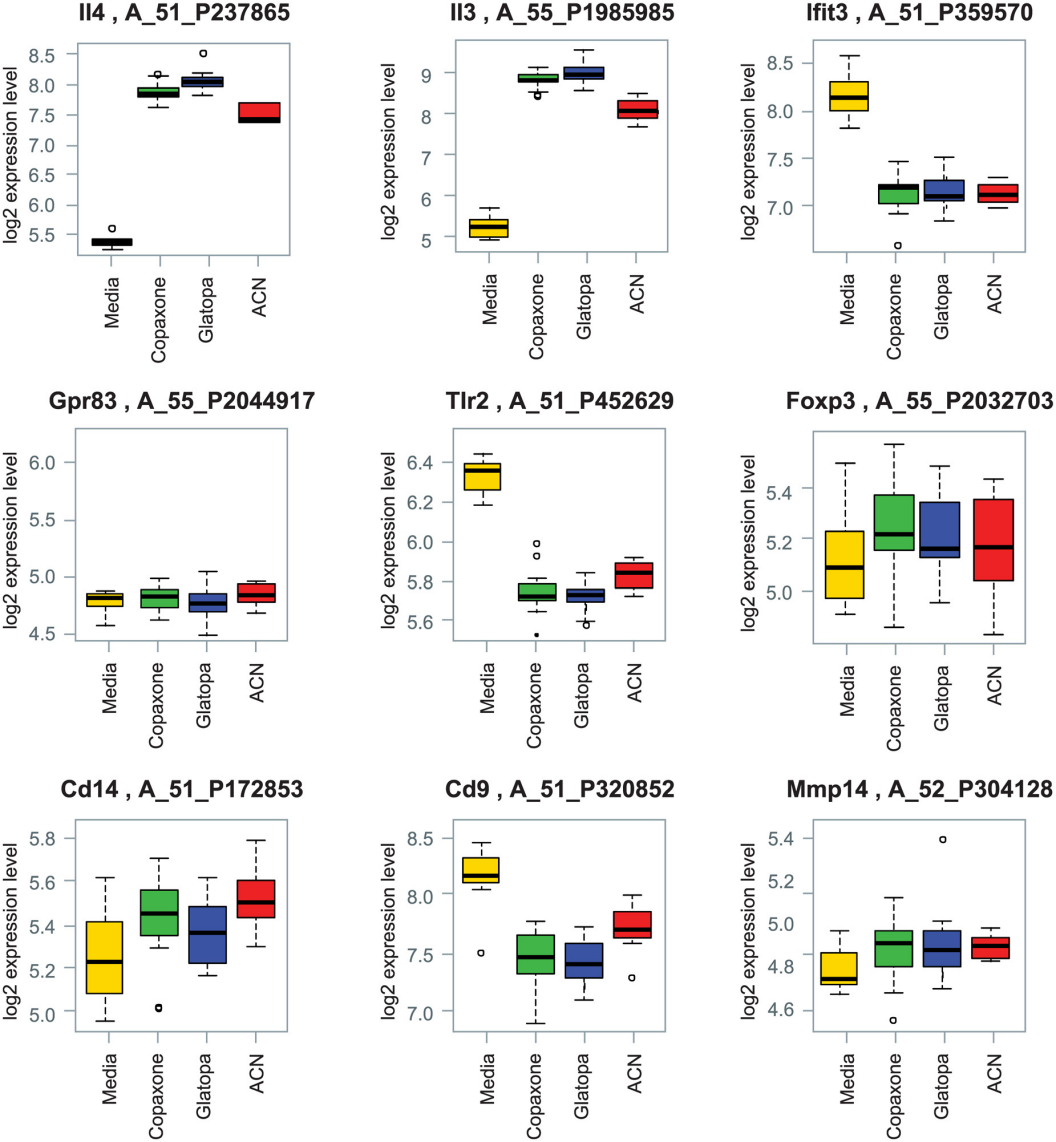
Box plots for gene expression changes for key Th2 cytokines IL-4 and IL-3 and additional genes related to immune cell function. No statistically significant differences between Glatopa and Copaxone were observed for any of these genes.

Two important Th2 cytokines, IL-3 and IL-4, were induced by Copaxone. These changes were similar to those seen with Glatopa (Table 4 and Fig 7). Several key immune cell genes (e.g., *FoxP3* and *GPR83*) reportedly regulated by GA in other biological test systems [29,35] were also examined; Fig 7 shows box plots of seven such specific genes (*IFT3*, *FOXP3*, *GPR83*, *CD14*, *TLR2*, *CD9*, and *MMP-14*). In the GA-responsive Th2-polarized T-cell test system, the expression of these genes was unchanged (*IFT3*, *FOXP3*, *GPR83*, *CD14*, and *MMP-14*) or decreased compared with media-only control. More importantly, no statistically significant differences were observed between the expression levels for any of these genes when Glatopa was compared with Copaxone.

## Discussion

The primary aim of this study was to assess the equivalence of the gene expression induced by Copaxone and the FDA-approved generic GA Glatopa using a robust, reproducible biological test system that was discriminatory and relevant to a mechanism of action of GA in patients with MS. To this end, a Th2 GA-responsive murine T-cell bank was generated and characterized, replicate samples of multiple lots of Glatopa and Copaxone were tested in this experimental system using a well-established whole-genome microarray technology platform. Numerous statistical (univariate and multivariate) methods were used to look for significant differences while controlling for false positives between the samples at the single gene level and the entire genome level. In parallel to testing of Glatopa and Copaxone, the nonequivalent glatiramoid molecule (ACN) was also tested to independently establish the ability of this experimental system to detect any potential differences. ACN is a glatiramoid that had the same molecular weight distribution and amino acid composition as GA; however, it was made by process conditions that generated a structurally nonequivalent mixture. Furthermore, given that Th2-cell polarization in humans is thought to be one of the main mechanisms of action of GA [4,8,10,17] and that many factors (including IL-4, IL-5, IFN-γ, and IL-10) observed to be stimulated in the system under study are also observed to be modulated by GA in humans [6,17], the Th2-polarized T cell experiment system used in this study is relevant to the mechanisms observed in humans for the treatment of relapsing forms of MS with GA. The variety of well-accepted statistical methods could not reject the hypothesis of equivalent gene expression profiles between Glatopa and Copaxone.

Other gene expression studies assessed the impact of glatiramoids on the expression of genes associated with MS [29,35]. They used the in vitro stimulation of murine T cells with various glatiramoids, including GA reference standards and drug products manufactured by Teva Pharmaceuticals [29,35]. Similar to the results from our study, Bakshi et al [29] found a significant difference in gene expression between the control (media only) and glatiramoid-treated cells. That study also identified significant differences in the expression of 98 genes when T cells were stimulated with a different and non-FDA—approved generic GA (Natco Pharma Ltd, Hyderabad, India) compared with Copaxone [29]. In the current study, no significant differences were found between Copaxone and Glatopa. Towfic et al [29,35] observed significant differences in expression of genes (FoxP3 and Gpr83) associated with Treg cells between Copaxone and non-FDA—approved generic GA (Natco), while the current studies did not find any changes in these genes independent of the test article used. Given that our test system utilized a population of Th2 polarized T cells generated by multiple rounds of GA stimulation, one likely explanation is that this cell population was primarily composed of memory T cells and not Treg cells. Thus the differences in our results and that of Towfic et al are most likely due to difference in experimental system. There also could be methodological differences in the studies; for example, the dataset by Towfic et al was generated in 18 different batches and thus required substantial correction, while our dataset was generated in a single batch.

Compared with our study, in which cells harvested from mouse lymph nodes were isolated 11 days after immunization and were further expanded from a CD4^+^ T-cell population, Bakshi et al [29] used cells harvested from mouse spleens 3 days after immunization with a single dose of Copaxone. The cell composition (primarily B and T cells) substantially differed from that of the lymph nodes. In addition, Bakshi et al did not perform any rounds of restimulation for selection of Copaxone-specific T cells, whereas our experimental system used cells expanded for 13 rounds of Copaxone stimulation, providing a significant enrichment of the GA-responsive T-cell population. Thus, it is conceivable that the response to various GA samples was more variable in the Bakshi study.

Although gene expression profiling is a useful tool in the analysis of mechanism of action of novel drugs, it can also be used as one of multiple methods used to assess equivalence of the effects of generic drugs. The current gene expression study was part of a much larger set of assays. Equivalence was established by determining sameness of starting materials, control of process, and equivalence of physicochemical, biological, and immunologic properties, including but not limited to multiple methods for amino acid composition, molar mass distribution, N- and C-terminal analysis, potency, T-cell biology, B-cell biology, APC biology, and animal models of the disease (e.g., experimental autoimmune encephalitis).

In conclusion, the use of gene profiling as part of a comprehensive set of analytical tools has furthered the demonstration of the equivalence of Glatopa and Copaxone. As expected, Copaxone induced substantial changes in gene expression in the test system. Changes observed included genes related to the mechanism of action of GA in MS. Differences in gene expression were found between Copaxone and a nonequivalent glatiramoid mixture. However, there were no significant differences in gene expression between Copaxone and Glatopa, as demonstrated through multiple statistical methods.
